# Etodolac transdermal cubosomes for the treatment of rheumatoid arthritis: *ex vivo* permeation and *in vivo* pharmacokinetic studies

**DOI:** 10.1080/10717544.2017.1326539

**Published:** 2017-05-23

**Authors:** Salwa Salah, Azza A. Mahmoud, Amany O. Kamel

**Affiliations:** 1 Department of Pharmaceutics and Industrial Pharmacy, Faculty of Pharmacy, Cairo University, Kasr El-Aini Street, Cairo, Egypt,; 2 Department of Pharmaceutical Technology, National Research Center, Dokki, Cairo, Egypt,; 3 Department of Pharmaceutics and Pharmaceutical Technology, Faculty of Pharmaceutical Sciences and Pharmaceutical Industries, Future University in Egypt, Cairo, Egypt, and; 4 Department of Pharmaceutics and Industrial Pharmacy, Faculty of Pharmacy, Ain Shams University, Abbassia, Cairo, Egypt

**Keywords:** Etodolac, transdermal, cubosomes, *ex vivo*, human bioavailability

## Abstract

In this study, transdermal etodolac-loaded cubosomes were developed in order to relieve patient pain and joints stiffness by providing stable etodolac concentration at the targeting sites through controlled drug delivery via the noninvasive skin route with more sustaining and less frequent dosing. Different ratios and percentages of poloxamer 407 and monoolein were used to formulate the cubosomes using emulsification and homogenization processes. The etodolac-loaded cubosomes showed particle size values ranging from 135.95 to 288.35 nm and zeta potential values ranging from −18.40 to −36.10 mV. All the cubosomes offered an encapsulation efficiency value of about 100% and showed drug loading capacity ranging from 1.28 to 6.09%. The *in vitro* drug release studies revealed a controlled drug release profile with a drug release rate up to 15.08%/h. Increasing poloxamer concentration in etodolac-loaded cubosomes resulted in nanoparticles with less particle size and faster drug release. The particles exhibited cubic and hexagonal shapes. The DSC and X-ray analysis demonstrated that the drug was encapsulated in the cubosomes bicontinuous structures in amorphous form. In addition, investigated cubosomes exhibited fast drug penetration through excited mice skin followed by slower drug penetration for up to 24 h. The pharmacokinetic study in human volunteers showed that the selected etodolac-loaded cubosomes enhanced the bioavailability of etodolac as compared to the oral capsules (266.11%) with evidence of longer half-life and higher MRT that reached 18.86 and 29.55 h, respectively. The etodolac-loaded cubosomes propose a promising system for treatment of arthritis simply through skin application.

## Introduction

Rheumatoid arthritis is a disease characterized by swollen, stiff and painful joints. This disease occurs because the body immune system starts to attack its own joints. There is no available cure for this disease; therefore, the treatment regimen aims to relieve patient pain and joints stiffness in order to improve patient ability to move. Patients suffering from rheumatoid arthritis need to use painkillers, like non-steroidal anti-inflammatory drugs (NSAIDs) such as cyclo-oxygenase-2 inhibitors or COX-2 s (Colebatch et al., 2011).

Etodolac is a non-steroidal anti-inflammatory selective cyclo-oxygenase-2 inhibitor drug and it is used in the treatment of rheumatoid arthritis (Jones, 1999). Unfortunately, etodolac therapy can cause general gastrointestinal disturbances and in some cases it can cause peptic ulcer and severe gastrointestinal bleeding (James and Reynolds, 1996). To avoid such complications of etodolac oral treatment, it is necessary to use an alternative route of administration. The key of successful treatment of inflammatory diseases using etodolac is to provide stable concentration of the drug in the blood for prolonged time with an effective low dose. Such treatment can be achieved by the application of transdermal etodolac dosage form.

The use of lipid-based dosage forms as lipophilic drug carrier has the ability to enhance the drug therapeutic efficacy (Bondi et al., 2003; Jain et al., 2005; Kwon et al., 2012; Salama et al., 2012a,b; El-Leithy et al., 2015). Cubosomes are lipid-based nanostructured aqueous dispersions in which the distinctive internalization of the dispersed particles is built up via a highly ordered spontaneous self-assembly process (Yaghmur and Glatter, 2009). Their name, cubosomes, reflect to their dispersion particle shape which consists of cubic liquid crystalline phase consisting of a highly twisted bicontinuous structures, two congruent non-intersecting water channels separated by continuous lipid bilayer (Patrick, 2009; Rizwan et al., 2009). Cubosomes can be prepared from biodegradable lipid materials like monoglycerides such as monoolein (MO). At room temperature, MO forms bilayers of continuous inverted cubic phase separating two intercrossing water channels. Cubosomes are produced from the emulsification of the cubic lipid phases in water (Hyde and Andersson, 1984; Caffrey, 1987; Mariani et al., 1988; Luzzati et al., 1993; Caboi et al., 1997). These cubosomes are potential drug nanocarriers since they are tailored for solubilizing higher amounts of amphiphilic, hydrophobic and hydrophilic drugs in their structures (Yaghmur and Glatter, 2009). Furthermore, cubosomes are highly biocompatible and bioadhesive nanoparticles (Landh and Larsson, 1996; Larsson, 2000).

Due to the similar cubic phase structure between the cubosomes and the stratum corneum, the cubosomes have penetration enhancing effect on the skin as the lipid part of the particles mix with the lipids of the stratum corneum and consequently fluidize the stratum corneum (Norlen and Al-Amoudi, 2004; Esposito et al., 2005). Furthermore, since cubosomes are known to be skin-adhesive (Spicer et al., 2002), these versatile drug nanocarriers can be promising drug carriers to be administrable by transdermal route (Pan et al., 2013).

In this regard, it would be a significant achievement to provide etodolac-based cubosomes to form biocompatible and bioadhesive transdermal dosage form as well as therapeutically effective nanocarriers leading to a significant decrease in etodolac side effects. Therefore, the purpose of this study was to prepare etodolac-loaded cubosomes, and to investigate the parameters influencing the properties of MO-based cubosomes. Furthermore, the ability of the cubosomes to act as transdermal carrier was *in vitro* and *in vivo* investigated.

## Materials and methods

### Materials

Etodolac was kindly gifted from European Egyptian Pharmaceutical Industries, Egypt and manufactured by Polpharma Pharmaceutical Works, Warsaw, Poland. Poloxamer 407 (Plx 407; Pluronic acid F127), phosphotungstic acid and MO were obtained from Sigma–Aldrich Chemie GmbH, Germany. Dialysis tubing cellulose membrane (molecular weight cutoff 12,000 g/mole) was purchased from Sigma Chemical Company (Sigma-Aldrich Corp., St. Louis, MO, USA). Poly(vinyl alcohol) (PVA; M.Wt. 22000 Da) was purchased from Sigma-Aldrich (Sigma-Aldrich Co., St. Louis, MO, USA). Potassium dihydrogen phosphate and disodium hydrogen phosphate were purchased from Sisco Research Laboratories Pvt. Ltd., Mumbai, India. HPLC grade of acetonitrile and methyl-t-butyl-ether were purchased from SDFCL – S. D. Fine Chemical Limited, Mumbai, India.

## Methods

### Preparation of etodolac-loaded cubosomes nanoparticles

Twelve cubosomes dispersions were prepared by emulsification of the cubic lipid phase consisting of MO and poloxamer 407 in water containing PVA (Morsi et al., 2014) as shown in Table 1. The cubosomes were prepared by melting MO and poloxamer 407 using hot plate kept at 60 °C (heating magnetic stirrer, Thermolyne Corp.). Then, the drug was dissolved in the molten mixture. After that, distilled water containing 2.5%w/w PVA, preheated at the same temperature, was added to the molten mixture under mechanical stirring at 500 rpm. Dispersions were maintained under stirring at room temperature for 2 h in order to solidify the lipid droplets. Afterwards, the dispersions were subjected to homogenization at 15,000 revolution per min at 60 °C for 1 min (Heidolph Homogenizers, Silent Crusher M, Germany). After cooling the preparations, the nanoparticles were maintained at room temperature in glass vials.

**Table 1.** Composition and characterization parameters values for etodolac loaded cubosomes nanoparticles.

Formula Code^a^	Plx^b^:MO^c^ Weight ratio	Plx^b^ (%w/w)	MO^c^ (%w/w)	Plx^b^ and MO^c^:Water Weight ratio	Particle size (nm)	Zeta potension (mV)	Loading capacity (%)	Viscosity (cp)
**F1**	–	0.00	4.76	1:20	223.45 ± 4.03	–18.40 ± 1.27	6.09 ± 8.11	3.14 ± 0.16
**F2**	1:6	0.68	4.08	1:20	182.85 ± 5.64	–33.95 ± 2.19	3.89 ± 5.25	3.14 ± 0.12
**F3**	1:4	0.96	3.80	1:20	178.70 ± 7.70	–31.75 ± 0.92	3.93 ± 5.24	3.14 ± 0.13
**F4**	1:2	1.58	3.18	1:20	142.35 ± 5.49	–31.85 ± 0.78	3.87 ± 5.27	3.66 ± 0.25
**F5**	–	0.00	9.10	1:10	288.35 ± 3.89	–32.70 ± 0.14	3.60 ± 4.58	3.66 ± 0.32
**F6**	1:6	1.30	7.80	1:10	184.65 ± 4.92	–32.35 ± 0.49	2.52 ± 2.59	3.14 ± 0.15
**F7**	1:4	1.82	7.28	1:10	162.35 ± 3.46	–31.00 ± 0.71	2.23 ± 2.83	4.71 ± 0.41
**F8**	1:2	3.04	6.06	1:10	135.95 ± 5.21	–33.95 ± 0.49	2.08 ± 2.50	4.71 ± 0.43
**F9**	–	0.00	16.66	1:5	248.10 ± 4.38	–28.20 ± 0.14	2.16 ± 2.45	6.80 ± 0.72
**F10**	1:6	2.38	14.28	1:5	219.50 ± 6.79	–33.60 ± 0.28	1.28 ± 1.75	75.86 ± 5.42
**F11**	1:4	3.34	13.32	1:5	197.90 ± 9.90	–36.10 ± 2.97	1.74 ± 2.06	80.57 ± 7.05
**F12**	1:2	5.56	11.10	1:5	162.5 ± 3.72	–32.70 ± 0.99	1.64 ± 1.95	85.58 ± 4.25

Etodolac was used in concentration of 2% w/w.

^a^ The aqueous solution contained 2.5%w/w poly(vinyl alcohol) was added to the formulation to get 100% w/w.

^b^ Plx: Poloxamer 407.

c MO: Monoolein.

### Characterization of the etodolac-loaded cubosomes dispersions

#### Determination of particle size and zeta potential for etodolac-loaded cubosomes nanoparticles

The average particle size of the cubosomes nanoparticles as well as their size distribution (polydispersity index; PDI) were measured by a particle analyzer (Zetasizer Nano ZS, Malvern Instruments, Malvern, UK) based on dynamic light scattering at angle of 173° and at a temperature of 25 °C. The cubosomes nanoparticles zeta potential values were also detected depending on the laser Doppler anemometry of the Zetasizer Nano ZS instrument (Malvern Instruments, Malvern, UK) and at a temperature of 25 °C.

#### Determination of encapsulation efficiency (EE%) for cubosomes nanoparticles

In order to quantify the drug content of cubosomes dispersions, accurate amount of filtered dispersion (Whatman filter paper No 41; pore size of 20–25 μm; UK) (Salama et al., 2016) was dispersed in acetonitrile and were vortexed for 10 min (Reax top, Heidolph, Germany). The drug content was then analyzed by LC-MS/MS method following the procedure reported below. The encapsulation efficiency (EE%) was estimated using the equation:

Encapsulation efficiency (%)= (Amount of encapsulated drug/Total amount of added drug)× 100

#### Determination of loading capacity for etodolac-loaded cubosomes nanoparticles

An accurate volume of cubosomes nanoparticles were filtered (Whatman filter paper No 41; pore size of 20–25 μm; UK) (Salama et al., 2016) and dried using freeze dryer (Christ freeze dryer, ALPHA 2-4 LD plus, Germany). An accurate weight of the dried cubosomes nanoparticles was then dispersed in acetonitrile and was vortexed for 10 min (REAX top, Heidolph, Germany). The drug content was then analyzed using LC-MS/MS method following the procedure reported below. The loading capacity (LC) for the cubosomes nanoparticles was calculated according to the following equation:

Loading capacity (%)=(Amount of encapsulated drug/Cubosomes weight) × 100

#### Determination of pH values for etodolac-loaded cubosomes nanoparticles

The pH values for the prepared etodolac-loaded cubosomes nanoparticles were measured using a Jenway bench pH meters (Model 3505, UK).

#### Rheological behavior of etodolac-loaded cubosomes nanoparticles

The rheological behavior of etodolac-loaded cubosomes nanoparticles was determined using cone and plate rheometer (Brookfield DV3THB cone/plate rheometer) at 25 ± 2 °C. The revolution per minute was increased linearly from 0.5 to 200 rpm. The behavior of the etodolac cubosomes nanoparticles was analyzed adopting different models.

#### *In-vitro* release of etodolac from cubososmes nanoparticles

The etodolac release from cubosomes nanoparticles were performed across dialysis cellulose membrane mounted on automated Hanson Franz diffusion cell system (MicroettePlus; Hanson Research, Chatsworth). The receptor compartment was filled with phosphate buffer saline (6.8 mL, pH 7.4) and the diffusion area was 1.767 cm^2^. The receptor medium was stirred at 200 rpm and maintained at temperature of 37 ± 0.5 °C in order to simulate human blood conditions. At different time intervals, samples were collected and were analyzed using LC-MS/MS following the method mentioned below. Release data were flitted to Korsmeyer–Peppas model (Korsmeyer et al., 1983; Peppas, 1985) in order to determine the drug release pattern. The drug release rate constant (k), time required for half of the drug to be released (*t*_50_) and the time required for 90% of the drug to be released (*t*_90_) were also determined.

#### Morphology of cubosomes nanoparticles

Selected cubosomes nanoparticles were morphologically observed by transmission electron microscope (Jeol, JEM-1230, Tokyo, Japan). The cubosomes nanoparticles were applied on a carbon-coated 300 copper grid and then they were stained using 2% phosphotungstic acid.

#### Differential scanning calorimetry (DSC)

Physical state of the selected cubosomes nanoparticles and their individual components as well as their physical mixtures were performed using a Shimadzu differential scanning calorimeter (DSC-50, Shimadzu, Japan). Samples were placed in standard aluminum pan and then they were heated at a constant rate of 5 °C/min under the atmosphere of nitrogen gas carrier in a temperature range from 20 to 400 °C.

#### X-ray diffraction (XRD)

X-ray diffraction patterns for the drug and selected formulations were analyzed using Philips-PW-1050 X-ray diffractometer operated at voltage (I) of 20 mA and current (U) of 40 kV using Ni filter and CuKa radiation (Holland).

#### *Ex vivo* skin permeation studies

*Ex vivo* etodolac permeation through fresh skin of newly born Albino mice (age 6 days or younger) was carried out using Franz diffusion cell adopting the same procedure followed in the release section. The skin of newly born mice were obtained from the Future University Labs, Cairo, Egypt. The drug steady state flux (Jss) was calculated from the slope of the linear portion for the plotted curve for the cumulative amount of etodolac permeated per unit area as a function of time (Elias et al., 1984). The permeability coefficient (kp) of etodolac across the skin from the investigated cubosomes nanoparticles was calculated as follows:

kp = Jss/C

where Jss is the drug steady state flux and C is the drug concentration in donor compartment.

## Pharmacokinetic study in healthy human volunteers

### Study design and subjects

A single-dose, three-period randomized cross-over design was adopted under fasting condition. Six healthy adult male volunteers participated in this study. Their mean age was 25.5 ± 2.5 years, mean height was 162.25 ± 9.3 cm, and mean body weight was 78.4 ± 6.9 kg. The purpose of the study was fully explained, and volunteers had given their written consents. The volunteers were instructed to cease from taking any drug, including over-the-counter, for 2 weeks prior to and during the study period. The study was conducted according to the guidelines of Good Clinical Practices (Baber, 1994) and the revised Declaration of Helsinki for bio-medical research (Association, 2001). The study protocol was reviewed and approved by the institutional review board of Genuine Research Center, Cairo, Egypt.

### Drug administration and sample collection

The volunteers were hospitalized at 9:00 p.m. and had a standard dinner in the clinical site. After an overnight fast (10 h), subjects were given a single oral dose of Etodolac 200 mg capsule (NDC: 51672401601, Apotex) or applied topically 500 μl from the selected etodolac formulas F3 and F4 containing 200 mg drug to the forearm skin of both arms using HILL TOP CHAMBER plain patch with 1 cm diameter according to a randomization plan. Volunteers were not allowed to eat or drink anything other than water until 4 h after dosing. A standard breakfast, lunch and dinner were then a served to all volunteers according to a standard time schedule. Between studies, the subjects were domiciliary with instruction. Food and caffeine containing beverages were not permitted over the entire course of study. The volunteers were under medical supervision at the study site, where they walked around or sat and were prohibited from strenuous activity until the 4 hr blood collection. Adverse events as well as abnormal laboratory values were reported and evaluated by the resident physician. At 0.0, 0.25, 0.5, 1.00, 1.50, 2.00, 3.00, 4.00, 6.00, 8.00, 12.00, 24.00 and 48.00 h after dosing 6 ml blood samples were drawn from the volunteers into evacuated heparinized glass tubes. Blood samples were then centrifuged for 10 min at 4 °C and 3500 rpm. Plasma was then transferred into 5 ml plastic tubes and stored at −20 °C. To complete the crossover design, the study was repeated in the same way after a washing out period of 15 days.

### Sample preparation

All frozen plasma samples were thawed at ambient temperature. Human plasma samples (0.5 ml) were placed in 7 ml glass tubes, and 100 μl of 200 ng/ml hydrochlorothiazide internal standard (IS) solution was added to each and vortexed for 1 min. Four ml methyl-t-butyl-ether (MTBE) was then added and samples were vortexed for 2 min. The tubes were then centrifuged for 10 min at 1790 g and the upper organic phases were transferred to clean glass tubes and evaporated to dryness at 40 °C using Vacufuge 5301 centrifugal vacuum concentrator (Eppendorf, Germany). The dry residues were dissolved in the mobile phase (200 μl) and vortexed for 1 min. Exactly, 20 μl was then injected in the apparatus using the autosampler.

### Analysis of plasma samples

An accurate, sensitive and selective LC-MS/MS method was developed and validated for determination of etodolac concentrations in plasma using chemicals and reagents of analytical grade. Hydrochlorothiazide internal standard (IS) stock solution was prepared by dissolving 10 mg in methanol and serially diluted with mobile phase to give a final working concentration of 200 ng/ml. A shimadzu Prominence (Shimadzu, Japan) series LC system equipped with degasser (DGU-20A3), solvent delivery unit (LC-20AB) along with auto-sampler (SIL-20 AC) was used to inject 20 μl aliquots of the processed samples on a Luna C (Phenomenex Inc, CA, USA) (50 × 4.6) mm, 5 μm particle size. All analysis was carried out at room temperature. The isocratic mobile phase (pH 4.5) composed of acetonitrile and (0.02 M) ammonium acetate buffer (70%, 30%, v/v) and 0.1% formic acid was delivered at a ﬂow rate of 0.50 ml/min into the mass spectrometer’s electrospray ionization chamber. Quantitation was achieved by MS/MS detection in negative ion mode for both etodolac and hydrochlorothiazide internal standard, using a MDS Sciex (Foster City, CA) API-3200 mass spectrometer, equipped with a Turbo ion spray interface at 400 °C. The ion spray voltage was set at -4500 V. The nebulizer gas was set at 14 psi, curtain gas at 25 psi, auxillary gas at 30 psi and collision gas at 11 psi. The compound parameters, namely, declustering potential, collision energy, entrance potential and collision exit potential were –41 V, –35 V, –6 V, –4 V for etodolac and –66 V, –53 V, –12 V, –4 V for hydrochlorothiazide (IS), respectively. The ions were detected in the multiple reaction monitoring mode, monitoring the transition of the m/z 285.9 precursor ion to the m/z 211.9 for etodolac and m/z 295.9 precursor ion to the m/z 78.0 for IS. The quadrupoles (Q1 and Q3) were set on unit resolution. The analytical data were processed using Analyst software (Version 1.4.2).

### Pharmacokinetic and statistical analysis

For each subject, etodolac plasma concentration–time data was analyzed by non-compartmental pharmacokinetic models using kinetica software (version 4.4.1). The peak plasma concentrations (*C*_max_) and the time to reach this concentration (*T*_max_) were directly obtained from the concentration–time profile. The area under the plasma concentration–time curve (AUC) from time zero to 48 h (AUC_0–t_) was calculated according to the linear trapezoidal rule. The terminal elimination rate constant (λz) was estimated by linear regression of the terminal portion of the ln (concentration)-time curve, and then the elimination half life was calculated. HVD values were calculated to evaluate any sustainment in etodolac release, where it is the time span during which the plasma concentrations is at least 50% of the *C*_max_ value (HVD_t50%Cmax_ which is the width of the plasma concentration profile at 50% of the *C*_max_) (Meier et al., 1974). R_D_ was calculated where it is the ratio between the HVD_t50%Cmax_ values of the transdermal formulations and the capsules. R_D_ is indicative of sustained release effect where a ratio of 1.5, 2 and >3 shows, low, intermediate and strong sustained release effect, respectively (Meier et al., 1974).

### Statistical data analysis

Data analysis was carried out using SPSS (Version 17.0). The results were expressed as the mean of three experiments ± standard deviation. One-way ANOVA test followed by post-hoc tests was used to detect significant differences between tested values at *p* < 0.05.

## Results and discussion

### Preparation of etodolac-loaded cubosomes nanoparticles

The etodolac cubosomal dispersion was prepared without the use of organic solvents which paves the road to ‘‘green’’ drug delivery systems based on lipid carriers. PVA aqueous solution was used as a steric barrier where it is adsorbed to the surface of the emulsion droplet which prevents the aggregation of emulsion droplets and stabilize them (Hans & Lowman, 2002). PVA forms small particles with uniform distribution. Moreover, it produces particles that are easily dispersed in aqueous medium (Sahoo et al., 2002). Stabilizers are usually incorporated in the cubosomal dispersion to modify its surface properties and hence impart stability. The stabilization of etodolac cubosomes nanoparticles by poloxamer 407 occurred *via* the adsorption of the polypropylene oxide copolymer's hydrophobic moieties into the outer surface of the cubosomes nanoparticles which resulted in the shielding of the inverted-type self-assembled lipid nanostructure from the surrounding aqueous medium, whereas the polyethylene oxide copolymer's hydrophilic moieties dangled in water (Larsson, 1989; Gustafsson et al., 1996; Gustafsson et al., 1997). Increasing the concentration of poloxamer in the cubosomes formulations allowed the formation of smaller droplets by increasing the interfacial stability of cubosomal nanoparticles.

### Characterization of the etodolac-loaded cubosomes nanoparticles

#### Determination of particles size and zeta potential

The particle size analysis for cubosomes nanoparticles loaded with etodolac showed that their particle size values are within the nano range (135.95–288.35 nm). As indicated from Table 1, it is evident that the particle size of cubosomes is indirectly proportional to the increase in poloxamer concentration. Upon reducing poloxamer concentration, larger size cubosomes nanoparticles were formed due to the reduced interfacial stability that resulted from insufficient amount of surfactant leading to aggregation of nanoparticles (Mainardes and Evangelista, 2005; Feczkó et al., 2011). The particle size distribution of the cubosomes nanoparticles indicated by the polydispersity index values ranged from 0.23 ± 0.01 to 0.43 ± 0.02 which was an acceptable range.

Zeta potential of the prepared cubosomal dispersion was studied to determine the surface charge of the nanoparticles which is important for predicting the long term stability of the colloidal dispersion. The high zeta potential values provide sufficient electric repulsion which in turn prevents particles aggregation (Pal et al., 2011). The results of zeta potential study shows that etodolac-loaded cubosomes nanoparticles carried a negative charge with mean values of –18.40 to –36.10. This might be due to the presence of the fatty acid, MO (Hundekar et al., 2014). Moreover, the surface negative charge may be due to the PVA hydroxyl group that was anchored on the surface of the cubosomes (Xu et al., 2009). Generally, adding poloxamer 407 to the cubosomal dispersion resulted in cubosomes with more negative charge values due to the interaction between poloxamer 407 hydroxyl ions with the aqueous medium (Rizwan et al., 2009).

Particle charge is important as Kohli and Alpar reported that only negatively charged particles could permeate through the skin via channels created by the repulsive forces between negatively charged skin lipids and particles (Kohli and Alpar, 2004).

#### Cubosomes nanoparticles encapsulation efficiency (EE%)

The formulated cubosomes nanoparticles were mainly composed of the lipophilic material MO and both poloxamer 407 and PVA surrounding the nanoparticles. Obviously, it is expected that cubosomes nanoparticles can carry and deliver lipophilic drugs that can dissolve in its inverted-type self-assembled lipid nanostructure. In order to compare the different formulations, the amount of drug incorporated in the nanoparticles was determined for each one. It was found that due to the strong affinity between etodolac and the MO in the cubosomes nanoparticles, it was ‘grabbed’ in the liquid crystal structure and thus the encapsulation efficiency values were ranged from 100.50 ± 1.71 to 107.83 ± 8.08%. Such high drug encapsulation efficiency is desirable as they can reduce the volume of dosage form required to achieve the desired therapeutic effect.

#### Loading capacity for cubosomes nanoparticles

High drug loading capacity means increased formula loading with drug to the other components leading to firmer inclusion of the drug inside the particle resulting in slower drug release. Etodolac-loaded cubosomes nanoparticles showed that the drug loading capacity ranged from 1.28 to 6.09%. The prepared cubosomes nanoparticles from MO only (F1, F5, and F9) resulted in nanoparticles with higher loading capacity compared to their counterparts prepared with poloxamer 407. This is due to the lower weight of the freeze dried cubosomes nanoparticles F1, F5 and F9, due to the absence of poloxamer in their composition, which resulted in higher ratio between the drug and the polymer.

#### pH values measurement for etodolac-loaded cubosomes nanoparticles

The pH of the cubosomal dispersion was measured in order to examine any possible skin irritation that could happen due to change in the skin pH upon *in vivo* application. The pH values of the prepared etodolac-loaded cubosomes were in the range of 5.38 ± 0.30 to 6.62 ± 0.25 which are within the acceptable nonirritant pH range (Barry, 1983; Hadgraft, 2001).

#### Rheological behavior for etodolac-loaded cubosomes nanoparticles

The flow properties of the formulations can affect the spreadability and the residence time of the formulation at the application site. The measure of changes in shear stress with shear rates can be used to determine whether the rheological behavior of the samples is Newtonian or Non-Newtonian. For tested formulations, as the shear rate is increased, the shear stress is linearly increased and thus the steady state shear viscosity exhibits an almost Newtonian flow behavior. The investigated systems showed viscosity values that ranged from 3.14 to 85.58 cp. Such low formulation viscosity can increase drug penetration into skin which leads to high pharmacological effect.

Increasing the amount of MO and poloxamer 407 in the preparation of the etodolac cubosomal dispersion led to the formation of more solid-like liquid crystal materials that increased the formulation viscosity.

#### Etodolac release from cubososmes nanoparticles

The release of etodolac from the prepared cubosomes was studied over 24 hr using dialysis cellulose membrane. The mechanism of the drug release from the investigated etodolac-loaded cubosomes followed Non-Fickain or anomalous transport where the rate of etodolac release from the cubosomes was governed by both drug diffusion and polymer relaxation (erosion) (Table 2). The drug release rate from the investigated formulations ranged from 1.68 to 15.08%/hr (Table 2) offering a greater potential for customized drug release pattern from cubosomes nanoparticles over a broader range of applications.

**Table 2.** Release parameter values for etodolac-loaded cubosomes nanoparticles.

		Release data according to Korsmeyer–Peppas model			
Formula code	Release efficiency (RE %)	*n*-value	Release rate (K; %/hr)	t_50_ (hr)	t_90_ (hr)
**F1**	16.21 ± 0.86	0.68 ± 0.05	6.86 ± 0.47	10.79 ± 0.04	19.40 ± 0.10
**F2**	19.35 ± 3.59	0.72 ± 0.02	8.47 ± 1.24	8.49 ± 0.94	15.09 ± 1.55
**F3**	25.72 ± 3.30	0.71 ± 0.06	10.68 ± 1.05	6.79 ± 0.13	12.09 ± 0.17
**F4**	32.58 ± 0.87	0.64 ± 0.06	15.08 ± 4.28	5.53 ± 0.96	9.80 ± 1.80
**F5**	12.66 ± 2.09	0.69 ± 0.09	5.87 ± 1.69	12.54 ± 0.94	22.42 ± 1.60
**F6**	16.72 ± 2.55	0.79 ± 0.03	5.41 ± 0.31	12.00 ± 1.03	21.36 ± 1.92
**F7**	22.54 ± 0.24	0.77 ± 0.01	6.74 ± 0.25	10.08 ± 0.20	17.83 ± 0.39
**F8**	21.23 ± 4.16	0.82 ± 0.16	5.53 ± 2.15	11.45 ± 1.36	20.23 ± 2.07
**F9**	11.41 ± 0.10	0.78 ± 0.00	3.30 ± 0.03	19.84 ± 0.18	35.37 ± 0.33
**F10**	4.62 ± 0.05	0.62 ± 0.02	2.23 ± 0.12	36.17 ± 0.91	65.13 ± 1.59
**F11**	5.43 ± 0.05	0.61 ± 0.02	2.65 ± 0.94	30.78 ± 0.78	55.49 ± 1.69
**F12**	5.22 ± 0.04	0.79 ± 0.03	1.68 ± 0.10	38.02 ± 0.79	68.19 ± 1.30

All the tested etodolac-loaded cubosomes revealed a controlled drug release profiles with no burst drug release demonstrating that MO provides release-retarding properties (figures not shown). They showed *t*_50_ values that ranged from 5.53 to 38.02 h and *t*_90_ values that ranged from 9.80 to 68.19 h. This low drug expulsion is presumably related to the entrapment of the lipophilic drug etodolac by the MO and the slow diffusion of the drug from the inner water channels in the cubic phases (Kwon et al., 2010).

Interestingly, while the cubosomes F1, F5 and F9 have high drug loading compared to other formulations, they did not promote drug release. This suggest that etodolac concentration gradient between the cubic phase and the surrounding aqueous phase medium was not the factor affecting drug release from the etodolac-loaded cubosomes. Thus it can be assumed that etodolac release from the cubosomes may be due to the diffusion of the drug from the inner water channels in the upper layers of cubic phases.

The release efficiency values for etodolac cubsomes nanoparticles containing only MO, F1 and F5, were significantly less (*p* < 0.05) than their counterparts containing both MO and poloxamer 407. Furthermore, It was observed that in cubosomes nanoparticles containing 1:20 or 1:10 weight ratio of poloxamer to MO there was an increase (*p* < 0.05) in their release efficiency values, generally, by increasing poloxamer concentration. This could be explained on the basis that etodolac release is governed by its partition between the existing phases. The nonionic surfactant, poloxamer 407, has the ability to solubilize the drug in the aqueous release medium. Thus, higher affinity of the drug to the *in vitro* release medium facilitates drug release from the cubosomes nanoparticles. Moreover, increasing poloxamer concentration in etodolac cubosomes nanoparticles resulted in cubosomes with less particle size and thus larger surface area for drug diffusion leading to faster etodolac release.

The viscosity of the preparation has been shown to be an important variable that could influence diffusivity of the drug from it. It was found that F10–F12 demonstrated lower drug release profiles compared to that for F9. This retardant effect of poloxamer can be explained by the slow diffusion of lipophilic drug through the high dense hydrophilic poloxamer 407 layer adsorbed around the MO.

Comparing the release data for the cubosomes formulations revealed that decreasing the amount of water used in cubosomes preparation from 95.24% w/w (F1–F4) to 90.90% w/w (F5–F8) and to 83.34% w/w (F9–F12) showed a significant (*p* < 0.05) decrease in drug release from the formulations. This may be due to increasing the amount of MO in the formulations which decrease the hydrophilicity of the particles inhibiting water penetration from the release medium, which in turn resulted in lower drug release. Furthermore, the release of etodolac from cubosomes with high poloxamer content may be retarded due to the formation of network between the poloxamers units which increased the viscosity (Table 1) and thus the penetration of the release medium into the cubosomes became difficult, moreover, it increases the drug diffusion path length.

It can be observed from the *in-vitro* drug release study that formulations, F3 and F4 showed the highest drug release within 24 h (81.43 and 89.19% at 24 h, respectively) and thus were selected for further investigations.

## Cubosomes nanoparticles morphology

**Figure 1.**
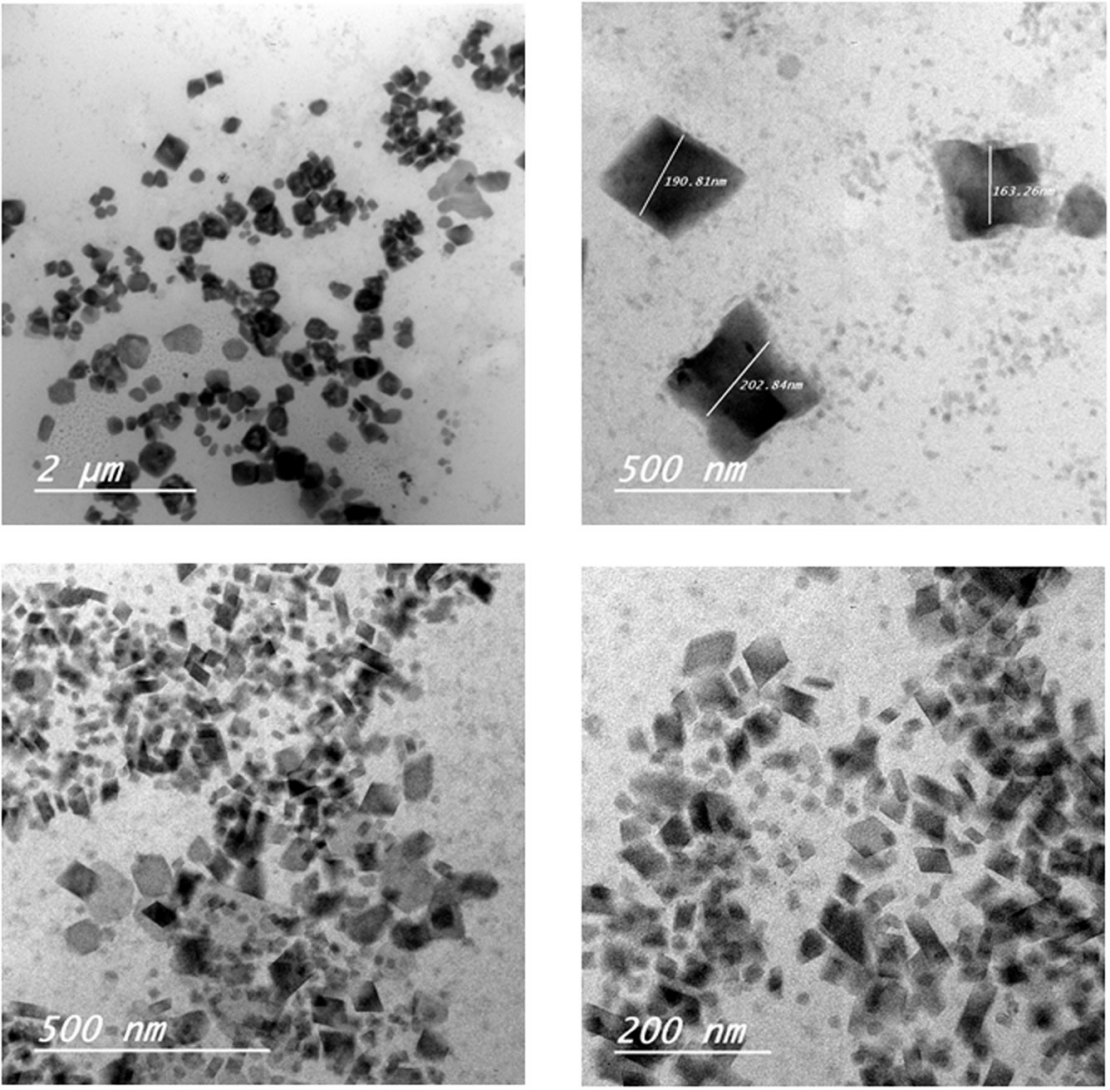
Transmission electron microscopy (TEM) images of etodolac cubosomes nanoparticles: a and b for F3 and c and d for F4.

Figure 1 shows TEM images taken for F3 and F4 formulations. The particles exhibited mostly cubic shape with zero mean curvature, however a small population of hexagonal vesicles was also found. The particles were in the nanorange of particle size and were well separated from each other. Cubosomes nanoparticles F4 showed smaller particles size compared to that for F3. This finding was verified before by particle size analysis and can be explained by the presence of larger amount of poloxamer 407 adsorbed on the cubosomes surface acting as a coating layer to stabilize the small surface of cubosomes nanoparticles.

## Differential scanning calorimetry (DSC)

**Figure 2.**
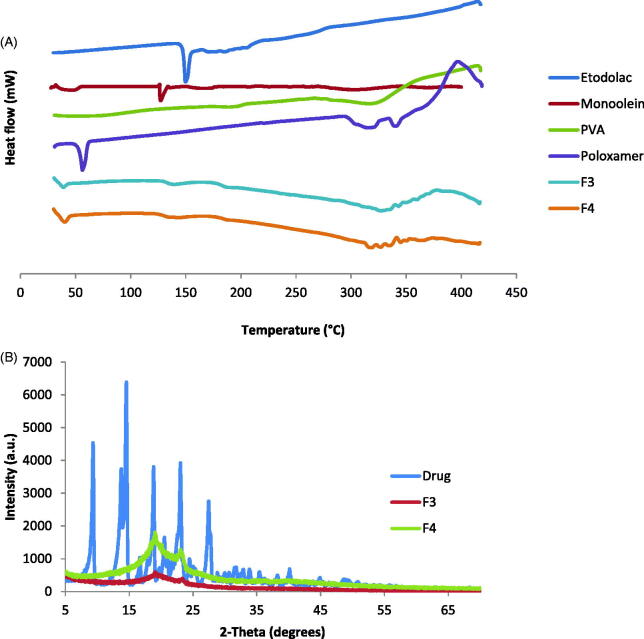
Physicochemical characterization for cubosomes nanoparticles: (A) DSC thermograms of (1) etodolac, (2) monoolein, (3) PVA and (4) poloxamer 407 as well as cubosomes formulations (5) F3 and (6) F4; (B) X-ray diffraction of etodolac as well as cubosomes formulations F3 and F4.

DSC is a powerful tool in evaluating the physicochemical drug-carrier interactions and powerful in detecting polymorphic modifications, therefore, DSC thermograms were studied for pure etodolac, MO, poloxamer, PVA and selected cubosomes formulations (Figure 2A). The DSC thermogram of pure etodolac, demonstrated a sharp endothermic peak at 149.75 °C corresponding to its melting temperature, such sharp peak indicates the crystalline state of the drug. Lipids like MO exhibit a common feature of having temperature-dependent phase transition. Lipids exist in a ‘‘gel’’ state below their transition temperature. An increase in temperature results in the transition from gel state to a liquid crystalline state (Ganem-Quintanar et al., 2000). Therefore, MO exhibited an endothermic peak at 45.03 °C due to its melting and further increase in temperature during the DSC study revealed a sharp endothermic peak at 127.30 °C corresponding to the melting of its liquid crystalline state. Poloxamer 407 showed endothermic peak at 56.26 °C due to its melting point and two endothermic peaks at 317.60 and 339.47 °C due to its decomposition. PVA sample showed two endothermic peaks at 188.99 and 316.49 °C due to its glass transition temperature and melting temperature, respectively (Joge et al., 2013).

In the investigated cubosomes formulations, small endothermic peaks were detected at 39.00 and 40.19 °C for F3 and F4, respectively. This lower shift of the MO endothermic peak could be due to the formation of the bicontinuous structures between the MO and water. The thermogram reveals also that the drug sharp peak disappeared, indicating its conversion to the amorphous state and indicating that the drug was molecularly encapsulated in the bicontinuous structures.

## X-ray diffraction (XRD)

The X-ray diffraction patterns of pure etodolac, F3 and F4 are shown in Figure 2(B). The diffraction peaks of etodolac showed specific sharp crystal peaks indicating its crystalline nature. F3 and F4 did not show the high intensity peaks of etodolac indicating that etodolac exists in the amorphous state and is no longer present as a crystalline material when incorporated into the bicontinuous structures. These results are in agreement with the DSC findings and allow the suggestion that the drug is encapsulated in the cubosomes nanostructure.

## *Ex vivo* skin permeation studies

Hairless mouse skin has been found to be an adequate, quantitative model for human skin in the investigation of chemical permeation enhancers (Kim et al., 1992, Li et al., 1997, Chantasart et al., 2004). This is due to that it is more easy to interpret the data from *ex vivo* permeation experiment using hairless mouse skin (Chantasart et al., 2004) due to the relatively constant lipid content of the stratum corneum for hairless mouse skin compared to the various lipid content of human skin (Raykar et al., 1988). Furthermore, the lipid composition of hairless mouse skin (Muller et al., 2002) is similar to that of human skin (Lampe et al., 1983). Therefore, in this study, hairless mouse skin was used to differentiate the between the permeation of etodolac from the tested cubosomes.

**Figure 3.**
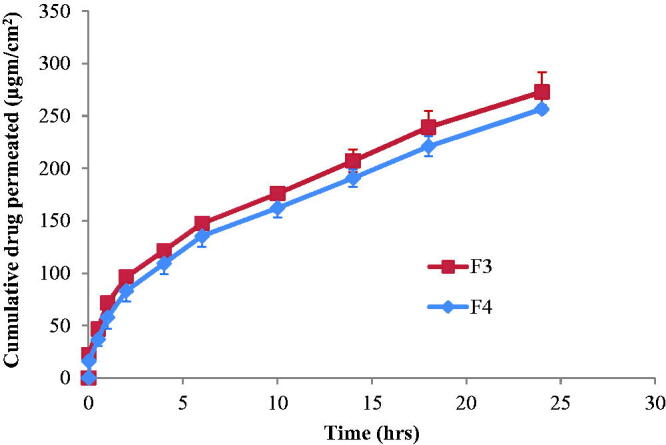
Permeation of etodolac from F3 and F4 cubosomes nanoparticles through excited mice skin.

The etodolac penetration from the investigated formulations, F3 and F4, through excited mice skin is demonstrated in Figure 3. It can be observed that there was no lag time in drug penetration through the skin from the investigated formulations. This might be due to the penetration action effect of both MO and poloxamer 407. MO can perturb the ordered structure of the skin by forming hydrophobic interaction with the skin lipid (Kwon and Kim, 2010). The nonionic surfactant, poloxamer 407, acts as penetration enhancer by emulsifying sebum, thereby enhancing the thermodynamic coefficient of the drugs (Maibach and Smith, 2005).

By fitting the data for etodolac penetration from F3 and F4 through the skin to Korsmeyer–Peppas model (Korsmeyer et al., 1983, Peppas, 1985), it was found that they have n-values less than 0.5 (0.36 and 0.40, respectively). This would indicate diffusion pattern for the drug penetration through the skin.

The drug penetration through the skin was characterized by two phases. The first phase was fast drug penetration at the first 2 h followed by slower drug penetration during the next investigated hours. The fast etodolac penetration through the skin may be due to the ability of the cubosomes to penetrate into the skin between corneocytes through the paracellular route (Subongkot et al., 2014). Further decrease in the drug penetration by time may be due to the formation of thin concentrated cubosomes layer that visually appeared on the skin. Such sustained drug diffusion pattern through the skin is also due to the ability of cubosomes nanoparticles to form a depot in the lipid part of the stratum corneum (Esposito et al., 2005). This may be due to the similarity between the structure of the nanoparticle cubic phase and the structure of the stratum corneum (Norlen and Al-Amoudi, 2004).

Comparing the penetration ability of etodolac from F3 to that from F4 revealed similar drug steady state flux values (7.92 ± 0.75 and 7.67 ± 0.24 μg/cm^2^/h, respectively) and permeability coefficient values of (0.0055 and 0.0053 cm/h, respectively) (*p* > 0.05). This is due to the fact that although F4 has less amount of the penetration enhancer MO compared to F3, but this decrease in MO amount was accompanied by an increase in the amount of the penetration enhancer poloxamer 407. Furthermore, both formulations had comparable *in vitro* drug release rate (*p* > 0.05) through cellulose membrane (*k* values of 10.68 ± 1.05 and 15.08 ± 4.28**%/**h for F3 and F4, respectively). Therefore, both formulations were chosen for *in-vivo* transdermal study on rats.

## Pharmacokinetic study of transdermal etodolac-loaded cubosomes nanoparticles

**Figure 4.**
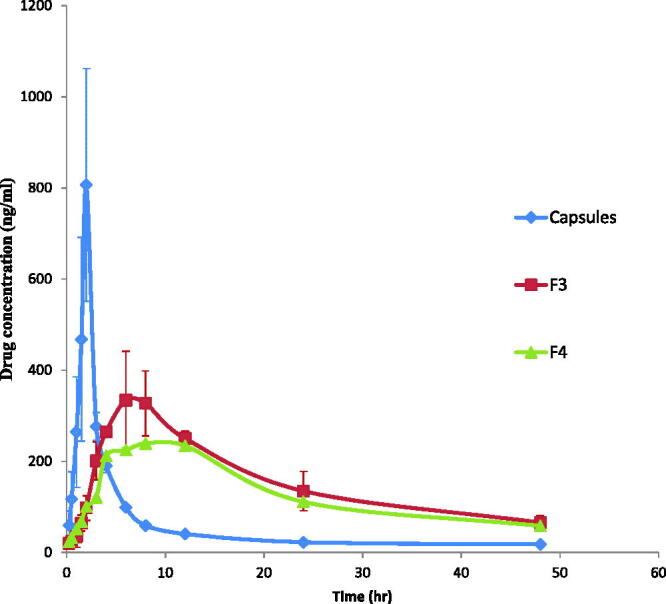
Etodolac mean plasma concentration (ng/ml) after oral administration of capsules and transdermal application of F3 and F4 cubosomal formulations to healthy human volunteers.

All human volunteers fully completed the study with no adverse reactions. The LC-MS/MS method of analysis has been validated and showed good linearity from 10 to 1000 ng/ml with acceptable within- and between-day reproducibility. The lower limit of etodolac quantiﬁcation in plasma was 0.1 ng/ml. The etodolac mean plasma concentration–time proﬁles following single oral dose administration of Etodolac 200 mg capsule (NDC: 51672401601, Apotex) and a single percutaneous application of F3 and F4 to six healthy human volunteers are shown in Figure 4.

The plasma concentration–time proﬁles as well as the calculated pharmacokinetic parameters showed that transdermal application of F3 and F4 cubosomes sustained the absorption of etodolac, expressed by the significant lower *C*_max_ values (340.66 ± 103.91 and 239.02 ± 3.72 ng/ml, respectively), significant delayed *T*_max_ values (8.00 and 8.00 h, respectively), significant prolonged t_1/2_ (17.32 ± 0.41 and 18.86 ± 0.53 h), significant higher MRT values (27.38 ± 0.82 and 29.55 ± 0.78 h, respectively) and significant higher HVD_t50%Cmax_ values (16.21 ± 2.97 and 19.88 ± 0.28 h, respectively) compared to that for the commercial etodolac capsule (*p* < 0.05) with *C*_max_, *T*_max_, t_1/2_, MRT and HVD_t50%Cmax_ values of 912.67 ± 12.08 ng/ml, 2.00 h, 6.00 ± 1.97 h, 11.68 ± 3.06 h and 1.01 ± 0.06 h. The ability of F3 and F4 in sustaining the absorption of etodolac was also proved by their high R_D_ values (16.05, 19.68, respectively) compared to that for commercial etodolac capsule (R_D_ value of 1). Moreover, the AUC_0-48 h_ values of F3 and F4 (7600.95 ± 1611.48 and 6331.07 ± 78.89 ng/ml.h, respectively) were signiﬁcantly (*p* < 0.05) higher than that of etodolac capsules (2856.27 ± 268.87 ng/ml.h). The higher value AUC_0-48 h_ of both F3 and F4 reﬂects their higher relative bioavailability of 266.11% and 221.66%, respectively compared to that for etodolac capsules (*p* < 0.05).

The low *T*_max_ and high *C*_max_ values following oral administration of etodolac are due to rapid absorption from the gastrointestinal tract. In contrast, the low *C*_max_ value and prolonged t_1/2_ after transdermal application of F3 and F4 are due to the barrier properties of the skin which led to an early accumulation of drug in the skin followed by its sustained release into the systemic circulation (Elshafeey et al., 2009). The higher MRT values following transdermal delivery of F3 and F4 compared to that for orally administered etodolac may be due to the interaction of the cubosomes with the stratum corneum lipids which might lead to the formation of etodolac cubosome depot that lead to continuous replenishment of drug in the systemic circulation by constant and controlled delivery of etodolac from the transdermal cubosomes. The significant increase in the relative bioavailability of the transdermal cubosomal dispersions is due to the combined effect of many factors. First, the unique structure of cubosomes offers high flexibility in transdermal drug delivery. Cubosomes structural organization is similar to that of the skin allowing them to be pressed through the pores of the stratum corneum and hence enhancing their transdermal delivery (Esposito et al., 2005). Second, GMO the major building unit of cubosomes is a well known penetration enhancer. It modifies the intercellular ordered structure of lipid bilayer in the stratum corneum, increases its fluidity and hence promotes the transdermal permeation of etodolac across the stratum corneum (Kwon et al., 2012). Third, the presence of poloxamer in cubsomes might have affected etodolac transdermal absorption. Poloxamer is composed of the hydrophilic ethylene oxide and the hydrophobic propylene oxide units which allows it to partition between the lipophilic mortar substance and the hydrophilic protein domains, hence penetrates deeply into the stratum corneum disrupting their lipid arrangement, increasing their fluidity and finally enhancing etodolac transdermal permeation (Yapar and Ýnal, 2012). Fourth, the anionic nature of the cubosomes contributed in enhancing etodolac permeation across the skin, where the negatively charged cubosomes permeated across the skin through channels produced by the repulsive force between the cubosomes and the skin (Rattanapak et al., 2012).

## Conclusion

In this study, transdermal cubosomes were developed. The developed delivery system is considered a key of successful treatment of rheumatoid as it provides controlled delivery of the drug in human *via* the noninvasive skin route with more sustaining, less frequent dosing and 266.11% relative bioavailability compared to the oral dosage form.
